# Investigating risk factors and antibiotic resistance of bloodstream infections causing *Klebsiella pneumoniae* in burn patients

**DOI:** 10.1097/MD.0000000000047037

**Published:** 2026-01-09

**Authors:** Lei Wang, Yanguang Li, Jian Zhang, Hongtao Xiao, Chengde Xia

**Affiliations:** a Department of Burn Surgery, Zhengzhou First People’s Hospital, Zhengzhou, Henan, China.

**Keywords:** antibiotic resistance, bloodstream infection, *Klebsiella pneumoniae*, risk factors, severe burns

## Abstract

This study aimed to investigate the risk factors associated with bloodstream infections (BSI) caused by *Klebsiella pneumoniae* (KP) and the antibiotic resistance of KP in patients with severe burns. This retrospective study included severe burn patients admitted to the Author’s Hospital. KP-induced BSI was confirmed by blood culture. The patients with confirmed KP-infection were placed in the KP-infection group and the remaining patients were included in the noninfection group. A total of 113 patients were included in the study, with 65 males. Among them, 23 patients were classified into the KP-infection group, and 90 patients were classified into the noninfection group. The multivariate logistic regression analysis found that total burn area (OR = 2.674; 95% CI: 1.265–7.164, *P* = .034), concurrent inhalation injury (OR = 3.295; 95% CI: 1.165–6.265, *P* = .026), third-degree burn area (OR = 2.834; 95% CI: 1.155–6.954, *P* = .012), and catheter indwelling time (OR = 3.169; 95% CI: 1.281–7.838, *P* = .009) were the independent risk factors for KP-dominated BSI in burn patients. All 23 KP strains from blood cultures were 100% resistant to ciprofloxacin, cefazolin, and piperacillin/tazobactam, 75.22% resistant to amikacin, 66.32% to cefoperazone/sulbactam, 60.87% to imipenem/cilastatin, and 60.87% to meropenem, while showing low resistance to tigecycline (4.35%) and polymyxin B (0.00%). Total burn area, concurrent inhalation injury, third-degree burn area, and catheter indwelling time were significant risk factors for BSI caused by KP in patients with severe burns. KP strains were highly resistant to multiple antibiotics, necessitating careful selection of susceptible antibiotics based on their resistance profiles.

## 1. Introduction

Each year, more than 67 million individuals worldwide suffer from burns, resulting in up to 3 million deaths, making it a significant cause of mortality due to fire/other accidents.^[1–3]^ Burns can cause extensive skin damage, fluid loss, and a hypermetabolic state in the body.^[4]^ The compromised natural immune barrier of the skin results in hospital-acquired infections.^[3,5]^ Patients with extensive skin barrier damage due to severe burns need prolonged intravenous access for fluid replacement. Decreased patient immunity and inadequate disinfection in the hospital contribute to high bloodstream infections (BSI). Catheter-related bloodstream infections (CRBSI) also occur in these patients.^[6–8]^ BSI, including CRBSI, prolongs hospitalization and increases mortality in patients with severe burns.^[9]^ Patients with burns trauma are at high risk of infections with multidrug-resistant bacteria and fungi, posing substantial challenges for the treatment.^[10]^

*Klebsiella pneumoniae* (KP), a common opportunistic pathogen in hospital-associated infections, is normally found in the human upper respiratory tract and intestines. A previous retrospective study investigated blood culture-positive sepsis in burn patients and found KP in blood cultures.^[11]^ It primarily causes pulmonary infections, urinary tract infections, and BSI, including CRBSI.^[12]^ It ranks second among Gram-negative bacteria detected in hospital-acquired infections, following *Escherichia coli*.^[13]^ It is difficult to treat and they are easy to spread, causing a high mortality rate.^[14]^

KP infections present significant treatment challenges due to their high levels of antibiotic resistance. Previous studies found that KP strains often produce extended-spectrum beta-lactamases and carbapenemases, which confer resistance to a broad range of beta-lactam antibiotics, including penicillins, cephalosporins, and carbapenems.^[15,16]^ This resistance limits the effectiveness of commonly used antibiotics, necessitating the use of more potent and often more toxic alternatives, such as colistin and tigecycline.^[17]^ Furthermore, the rapid spread of multidrug-resistant KP strains within healthcare settings exacerbates the difficulty in controlling infections and increases the risk of outbreaks.^[18]^ The high mortality rate associated with KP infections underscores the urgent need for effective infection control measures and the development of new antimicrobial agents.^[19]^

Therefore, this study aimed to identify the risk factors for KP-induced BSI in patients with severe burns and examine the isolates’ antibiotic resistance.

## 2. Methods

### 2.1. Study design and patients

**Figure 1.**
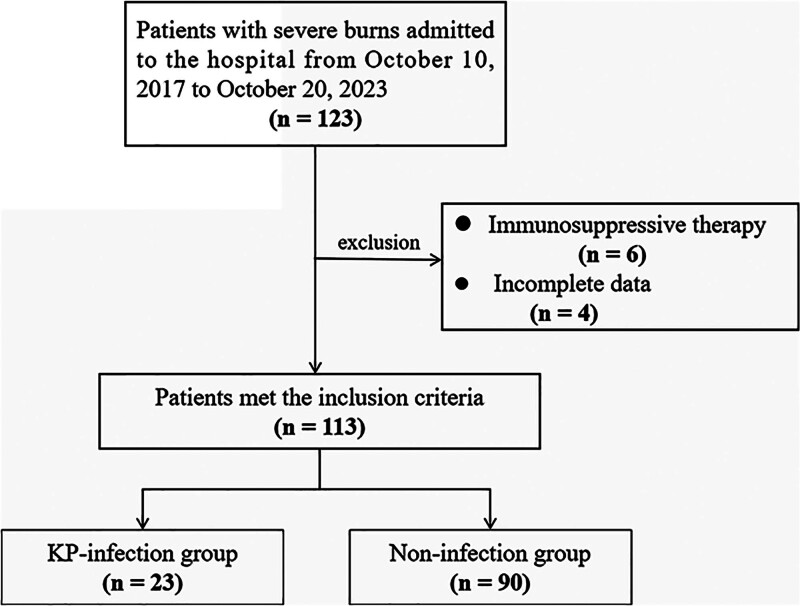
Screening flowchart for eligible patients.

This retrospective study included patients with severe burns admitted to the Author’s Hospital. The dates when data were accessed for research purposes from October 10, 2017, to October 20, 2023. The inclusion criteria were as follows: patients with severe burns which were defined as a total burn area of >30%, burn depth of second-degree or greater, with or without inhalation injury^[20]^; patients were diagnosed with BSI according to the “*Diagnostic Criteria and Treatment Guidelines for Burn Infections of Chinese Medical Association (2012 Edition*)”^[21]^; patients who were >18 years old; patients with complete clinical data. The exclusion criteria were as follows: patients who used immunosuppressive drugs within the past 6 months; patients who had simultaneous malignant tumors. KP-induced BSI was confirmed by blood culture. The flow chart for patient inclusion and exclusion is detailed in Figure 1. The patients with confirmed KP-infection were placed in the KP-infection group and the remaining patients were included in the noninfection group. The study protocol complied with the “Helsinki Declaration” in accordance with the Declaration of Helsinki (2000) issued by the World Medical Association. This study was approved by the Author’s Hospital. Informed consent was waived due to the retrospective nature of the study. The date of accessing the data for study purposes was February 16, 2024.

### 2.2. Therapeutic regimen

All patients received standard comprehensive burn care, including early fluid resuscitation for shock management, prophylactic measures to prevent early complications, anti-infective therapy, staged surgical debridement and autologous skin grafting, as well as enteral or parenteral nutritional support. Upon diagnosis of KP-induced bloodstream infection, targeted antibiotic therapy was administered based on antimicrobial susceptibility testing. In cases of carbapenem-resistant KP bloodstream infection, combination regimens based on tigecycline or polymyxin B were used.

### 2.3. Data collection

Demographic and clinical data were collected from the medical records, and the data included gender, age, cause of burns, total burn area, third-degree burn area, presence of inhalation injury, mechanical ventilation use, catheter placement site, and catheter indwelling time. The severity of burns was classified as follows: burns with 30 to 50% total burn area were classified as severe burns and burns with >50% total burn area were classified as extremely severe burns.^[20]^

The VITEK-2 compact automated microbial identification system was used to identify KP isolates and test their susceptibility/resistance to antibiotics, following the guidelines established by the Clinical & Laboratory Standards Institute (CLSI).^[22]^ The antibiotics included in the testing were ciprofloxacin, cefazolin, piperacillin/tazobactam, amikacin, cefoperazone/sulbactam, imipenem/cilastatin, meropenem, tigecycline, and polymyxin B.

### 2.4. Statistical analysis

The collected data were analyzed using SPSS 21.0 (IBM, Armonk). Categorical variables were expressed as n (%) and compared using the χ^2^ test. Univariate and multivariate logistic regression analyses were performed to identify the risk factors for KP-induced BSI in patients with severe burns. Variables with *P*-values <.05 in the univariate analysis were included in the multivariate logistic analysis. A 2-sided *P*-value of <.05 was considered statistically significant.

## 3. Results

A total of 113 patients were included in the study, of whom 65 were male. Among them, 23 patients were classified into the KP-infection group, while the remaining 90 patients were in the noninfection group. There were no significant differences in gender, age, cause of burns, mechanical ventilation cases, catheter placement sit, comorbidities (hypertension and diabetes) between the KP-infection and noninfection groups (all *P* > .05). However, patients in the KP-infection group had significantly larger total burn areas, higher rates of concurrent inhalation injuries, greater third-degree burn areas, and longer catheter indwelling times than patients in the noninfection group (*P* < .05) (Table 1).

**Table 1:** Univariate analysis of KP-induced BSI in patients with severe burns.

	KP-infection group (n = 23)	Noninfection group (n = 90)	*P*-value
Gender, n (%)			
Male	15 (65.2)	50 (55.6)	.403
Female	8 (34.8)	40 (44.4)	
Age			
≥40 yr	12 (52.2)	48 (53.3)	.921
<40 yr	11 (47.8)	42 (46.7)	
Cause of burns			
Thermal burns	15 (65.2)	65 (72.2)	.510
Other burns	8 (34.8)	25 (27.8)	
Severity of burns (%)			
Severe burns	2 (8.7)	42 (46.7)	.029
Extremely severe burns	21 (91.3)	48 (53.3)	
Third-degree burn area (%)			
<10	5 (21.7)	45 (50.0)	.015
≥10	18 (78.3)	45 (50.0)	
Concurrent inhalation injury			
Yes	16 (69.6)	52 (57.8)	.037
No	7 (30.4)	38 (42.2)	
Mechanical ventilation (cases)			
Yes	17 (73.9)	60 (66.7)	.506
No	6 (26.1)	30 (33.3)	
Catheter placement site			
Internal jugular vein	5 (21.7)	16 (17.8)	.904
Subclavian vein	8 (34.8)	34 (37.8)	
Femoral vein	10 (43.5)	40 (44.4)	
Catheter indwelling time (d)			
3–7	4 (17.4)	36 (40.0)	.043
>7	19 (82.6)	54 (60.0)	
Hypertension			
Yes	9 (39.1)	24 (26.7)	.241
No	14 (60.9)	66 (73.3)	
Diabetes			
Yes	8 (34.8)	19 (21.1)	.170
No	15 (65.2)	71 (78.9)	

Severe burns: burns with 30%–50% total burn area; extremely severe burns: burns with > 50% total burn area.

BSI = bloodstream infections, KP = *Klebsiella pneumonia*.

**Figure 2.**
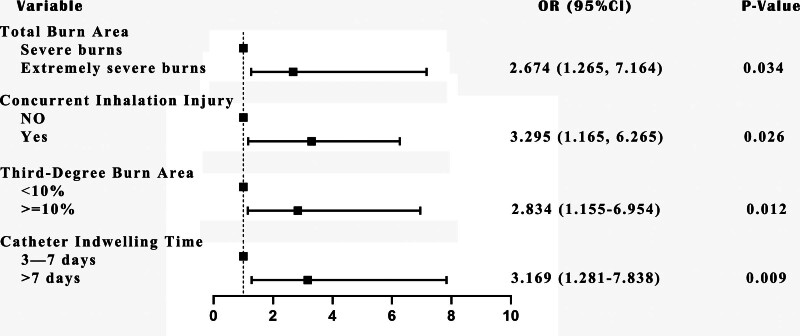
Forest plot of independent risk factors for KP-related bloodstream infection in severe burn patients. KP = *Klebsiella pneumonia*.

The multivariate logistic regression analysis found that total burn area (OR = 2.674; 95% CI: 1.265–7.164, *P* = .034), concurrent inhalation injury (OR = 3.295; 95% CI: 1.165–6.265, *P* = .026), third-degree burn area (OR = 2.834; 95% CI: 1.155–6.954, *P* = .012), and catheter indwelling time (OR = 3.169; 95% CI: 1.281–7.838, *P* = .009) as the independent risk factors for KP-dominated BSI in burn patients (Table 2). These findings are further illustrated in the forest plot (Fig. 2), which visually summarizes the estimated odds ratios and their 95% confidence intervals derived from the multivariate logistic regression model.

**Table 2:** Multivariate logistic regression analysis of KP-induced BSI in patients with severe burns.

Risk factors	OR	95% CI	*P*-value
Total burn area	2.674	1.265–7.164	.034
Concurrent inhalation injury	3.295	1.165–6.265	.026
Third-degree burn area	2.834	1.155–6.954	.012
Catheter indwelling time	3.169	1.281–7.838	.009

BSI = bloodstream infections, KP = *Klebsiella pneumonia*.

According to the analysis of antibiotic resistance in 23 KP strains, all strains exhibited resistance to ciprofloxacin, cefazolin, and piperacillin/tazobactam (all 100.00%). The resistance rates were relatively higher for amikacin (17, 75.22%), cefepime (15, 66.32%), imipenem/cilastatin (14, 60.87%), and meropenem (14, 60.87%). In contrast, the resistance rates to tigecycline (1, 4.35%) and colistin (0, 0.00%) were lower (Table 3).

**Table 3:** Antibiotic resistance of KP strains.

Antibiotic	Number of resistant strains	Resistance rate (%)
Ciprofloxacin	23	100.00
Cefazolin	23	100.00
Piperacillin/tazobactam	23	100.00
Amikacin	17	75.22
Cefoperazone/sulbactam	15	66.32
Imipenem/cilastatin	14	60.87
Meropenem	14	60.87
Tigecycline	1	4.35
Polymyxin B	0	0.00

KP = *Klebsiella pneumonia*.

All 113 patients included in the study achieved clinical cure and were successfully discharged from the hospital, including those with KP bloodstream infections. No in-hospital mortality was observed in either group.

## 4. Discussion

This study identified total burn area, concurrent inhalation injury, third-degree burn area, and catheter indwelling time as the independent risk factors for KP-dominated BSI in burn patients. All KP strains were absolutely resistant to ciprofloxacin, cefazolin, and piperacillin/tazobactam, highly resistant to amikacin, cefepime, imipenem/cilastatin, and meropenem, and not resistant to tigecycline and colistin. These findings highlight the need for careful monitoring and tailored antibiotic therapy in burn patients to effectively manage infections caused by KP.

BSI rate greatly varies in burn patients. A previous investigation^[23]^ found that 56 out of 87 patients with severe and extremely severe burns developed BSI, with an infection rate of 67.8%. In another study by Zhang et al,^[24]^ 86 out of 307 patients with extremely severe burns developed BSI, with an infection rate of 28.01%. In the current study, 23 out of 113 patients with severe burns developed KP-dominated BSI, resulting in an overall infection rate of 22.33%. This variation may have been due to the heterogeneity of the clinical characteristics of severe burn patients.

Similar to other studies,^[25,26]^ third-degree burn was a risk factor for KP-dominated BSI in the current study. Third-degree burns severely damage the skin and significantly affect its barrier function and immune response, disseminating KP in the blood and other organs.^[27,28]^ Consistent with a previous study,^[25]^ the total burn area was identified as a risk factor for BSI in the present study. As the total burn area increases, the severity of BSI also increases in burn patients.

Burn patients often require substantial fluid replacement through intravenous catheterization.^[29]^ However, improper catheter placement, inadequate catheter disinfection, and frequent removal and insertion can lead to catheter-related BSI in severe burn patients.^[30]^ Previous studies associated long catheter indwelling time with BSI.^[31,32]^ Similarly, this current study identified catheter indwelling time as one of the risk factors for KP-dominated BSI.

Bloodstream infection often causes mortality in burn patients. KP is one of the most frequently isolated organisms in patients with BSI. KP is also found to be a common pathogen in pneumonia and urinary tract infections.^[33]^ KP ranked second among gram-negative bacteria in community-acquired infections in China in 2020.^[24,34]^ KP strains have increasingly become resistant to various β-lactam antibiotics and these resistant strains can jump from 1 host species to another.^[35,36]^ Therefore, understanding the resistance profile of KP in BSI is of significant clinical importance.

In this study, isolated KP strains were completely resistant to ciprofloxacin, cefazolin, and piperacillin/tazobactam. The resistance rates to amikacin (75.22%), cefepime (66.32%), imipenem/cilastatin (60.87%), and meropenem (60.87%) were also high. The resistance rates to tigecycline (4.35%) and colistin (0.00%) were relatively low, similar to previous studies.^[32]^ Since the carbapenem-resistant-KP (CR-KP) is on the rise, imipenem/cilastatin, meropenem, ciprofloxacin, and cephalosporins can no longer be considered the first-line antimicrobial agents. Surveillance of antibiotic resistance is critical for treating infections in these patients.

This study also has some limitations. Firstly, as a retrospective study, recall bias is an unavoidable limitation. Secondly, the assessment of burn severity based on the total burn area and depth may have had some inaccuracies due to potential measurement errors. Thirdly, culturing biological specimens and identification of KP strains may have lacked sensitivity, potentially leading to some strains being missed. This could be due to the low positivity rate of blood cultures and the possibility of false negatives. Fourthly, due to the retrospective nature of the study and the lack of preserved bacterial isolates, molecular detection of drug resistance genes was not conducted, which may have limited the detailed description of the mechanism of bacterial drug resistance. Lastly, a relatively small sample size may have limited the precision of the study’s interpretations.

In conclusion, the total burn area, concurrent inhalation injury, the extent of third-degree burns, and catheter indwelling time were influential factors of BSI by KP in severely burned patients. Several KP strains were resistant to multiple antibiotics. Susceptible antibiotics should be carefully selected based on the resistance profile. Targeted management of patients and proactive infection prevention strategies are crucial in reducing the incidence of KP-related infections.

## Author contributions

**Data curation:** Lei Wang, Yanguang Li, Jian Zhang, Hongtao Xiao, Chengde Xia.

**Formal analysis:** Lei Wang, Yanguang Li, Jian Zhang, Hongtao Xiao, Chengde Xia.
